# Supplementary material for: A hybrid multiscale model for predicting CAR-T therapy outcomes in solid tumors
Source: Sci Rep. 2026 May 23;16:23671. doi: 10.1038/s41598-026-50412-6 (PMC13424311; doi:10.1038/s41598-026-50412-6)
Supplement: Supplementary file 1 — Supplementary Material 1 [file 41598_2026_50412_MOESM1_ESM.pdf]

Supplementary Material

A Hybrid Multiscale Model for Predicting CAR-T Therapy Outcomes in Solid Tumors

Mohammad R. Nikmaneshi[1], Lance L. Munn.[1,*]

[1] Department of Radiation Oncology, Massachusetts General Hospital and Harvard Medical School, Boston, MA 02114, USA

[*]Correspondence and requests for materials should be addressed to **L.L.M.** (email: lmunn@mgh.harvard.edu).

CONTENTS

Supplementary Data and Figures 3

1. Multi-Scale model of tumor-immune interaction 3

1.1 Molecular scale 3

1.1.1 Cellular respiration species- oxygen, glucose, and CO_2: 5

1.1.2 tumor-induced paracrine signals for T cells: 5

1.1.3 vessel development and remodeling: 6

1.2 Cellular Scale 8

1.2.1 Vitality of cancer and T cells 8

1.2.2 Endothelial cells 9

1.2.3 T cells and their interaction with ECs and cancer cells 9

1.3 Tissue Scale 13

1.3.1 development of tumor tissue and neo-vessel pathways 13

1.3.2 Vessel growth and remodeling 14

1.3.3 Fluid dynamics of the TME 15

2. Initial and boundary conditions 16

3. Assumptions and limitations 17

4. T cell distribution in the TME: 17

Correlation between HF and tumor regression: 19

Table S1. The parameters used for computational results of the mathematical model. 21

Bayesian Posterior Analysis of Calibrated CAR-T Parameters: 24

References: 25

Supplementary Data and Figures

1. Multi-Scale model of tumor-immune interaction

Computational recapitulation of tumor therapy, especially cell therapies such as CAR-T, requires a comprehensive mathematical model able to simulate important dynamics of the TME, including spatiotemporal distributions of biochemical and biomechanical factors, relationships between different spatial scales, including molecules, cells, and tissue. The model should also reproduce morphological heterogeneity of tumor growth and vasculature to properly analyze cell and nutrient distributions. Continuous, discrete and hybrid continuous-discrete models have been previously developed to simulate the TME. Continuous models can predict spatiotemporal distributions of biomolecules within the TME but ignore the morphological heterogeneity [1-5]. Discrete models can be used to analyze tumor vascularization and growth but don't explicitly consider transport of biomolecules, or their dynamic distributions [6-8]. Hybrid models combine the advantages of discrete and continuous models and are able to accurately recapitulate many aspects of TME dynamics and heterogeneities [3,9-14]. Our three-dimensional multi-scale mathematical models of the TME combine discrete and continuous methods to simulate the dynamics of tumor growth, angiogenesis and transport [14-17]. Here, we developed a 3D multiscale hybrid model of the tumor–immune microenvironment that incorporates both intravascular and interstitial T cell interactions. This model enables quantitative and qualitative characterization of T cell distribution within tumors through a parameter we define as "Tumor Hotness," and it further predicts cancer response to CAR-T therapy under varying environmental conditions and infusion strategies.

1.1 Molecular scale

The time-dependent concentration of each species in the 3D tumor microenvironment, c_i, is governed by Eq. 1, which includes convection by interstitial fluid flow, molecular diffusion, and a reaction term, *Ri*. The vascular compartment can be a source or sink for a given soluble species, represented as *Si*:

$$\frac{\partial c_i}{\partial t} + \nabla.(r_f u_{ins} c_i) = D_i \nabla^2 c_i + R_i + S_i \qquad (1)$$

D_i is the diffusion coefficient of species i, $\boldsymbol{u_{ins}}$ is the interstitial fluid flow (IFF) velocity, and r_f is the retardation factor defined by the ratio of the solute velocity to the interstitial fluid velocity. Species: $i = G$ *(glocuse),* o_2 *(oxygen),* co_2 *(carbon dioxide), v (VEGF), a1 (ang-1), a2 (ang-2), m (MMPs), e(ECM), col (Collagen), SF (suppressiv factors), AF (Immune attractive factor).*

<u>Vessels as a source or sink for soluble species</u>: For transvascular exchange of plasma and soluble species, we use a modified pore model to consider the convectional and diffusional transmigration of particle across the angiogenic vessels [14,18-22]:

$$S_i = (1-\sigma_{d,i})S_V\left(\frac{d_v}{d_c}\right)\left[L_p\left(p_{lum}-p_{ins}-\sigma_v(\pi_{lum}-\pi_{ins})\right)\right]c_{p,i} + \kappa_i S_V\left(\frac{d_v}{d_c}\right)(c_{p,i}-c_i)\frac{P_i}{e^{P_i-1}} \tag{2}$$

In this model, $\sigma_{d,i}$ defined in Eq. 3, is a function of particle size of solute, d_i, and pore size of porous media, d_p, is colloid osmotic (oncotic) reflection coefficient for solute into plasma and $\sigma_v$is average oncotic reflection coefficient of plasma proteins, $d_v$is angiogenic neo-vessel diameter,$d_c$is the neo-vessel characteristic diameter, S_V surface area per unit volume for transvascular exchange, p_{lum} is intravascular blood pressure, $p_{ins}$is interstitial fluid pressure (IFP), π_{lum} and π_{ins} are respectively oncotic pressures of the intravascular plasma and interstitial fluid, κ_i is permeability coefficient of the neo-vessel wall, L_p is the hydraulic conductivity of the neo-vessel wall, which is defined in Eq. 40.

P_i defined in Eq.4 is the transvascular Peclet number -- the ratio of convection to diffusion across the neo-vessel wall, $P_i = (1-\sigma_{d,i})\ (U_{TFF}\, t_v)/D_{t,i}$. U_{TFF} is the transvascular fluid flow velocity (transvascular fluid flow, Q_{TFF}, divided by surface area of vessel, A_v), t_v is vessel wall thickness and $D_{t,i}$ is diffusion coefficient of species across the vessel wall ($D_{t,i} = D_i$). If we replace $U_{TFF} = Q_{TFF}/A_v$ by Eq. 39 and then describe the permeability coefficient of the neo-vessel wall, $\kappa_i = D_i/t_v$, the transvacular Peclet number is governed by Eq. 4.

In Eq.2,$c_{p,i}$ the plasma concentration of species is assumed to be constant because the ratio of venous to arterial plasma concentration of species is close to 1 [14,18].

$$\sigma_{d,i} = \left(1-\left(1-\frac{d_i}{d_p}\right)^2\right)^2 \tag{3}$$

$$P_i = (1-\sigma_{d,i})\left[\frac{L_p}{\kappa_i}\left(p_{lum}-p_{ins}-\sigma_v(\pi_{lum}-\pi_{ins})\right)\right] \tag{4}$$

The relationship between vessel wall pore sizes of normal tissue (stroma), d_p^s, and tumor tissue, d_p^t, is derived via scale-up of the Kozeny-Carman relation as $d_p^s/d_p^t = (L_p^s/L_p^t)^{1/6}$. As shown in Table S1, we use a constant value for the pore size of intratumoral vessel walls, and calculate the pore size of the vessel wall in normal tissue based on this relationship.

Reaction models for molecular scale species, Ri

1.1.1 Cellular respiration species- oxygen, glucose, and CO_2:

Assumed to be a function of cellular vitality, the consumption of glucose and oxygen as well as the production of CO_2 by cells (cancer and T cells) are related by the stoichiometry of the cellular respiration reaction according to:

$$\begin{aligned}&CellularRespiration\ (Complete\ Oxidation\ of\ Glucose\ (OXPHOS)):\\&C_6H_{12}O_6 + 6O_2 \rightarrow 6CO_2 + 6H_2O + ATP\end{aligned} \tag{5}$$

$$R_G = -\underbrace{\frac{1}{6}k_m\,\gamma_0 \upsilon}_{consumption} - \underbrace{\varepsilon_G c_G}_{natural\ decay}$$

$$R_{o_2} = -\underbrace{k_m\,\gamma_0 \upsilon}_{consumption} - \underbrace{\varepsilon_{o_2} c_{o_2}}_{natural\ decay}$$

$$R_{co_2} = \underbrace{k_m\,\gamma_0 \upsilon}_{production} - \underbrace{\varepsilon_{co_2} c_{co_2}}_{natural\ decay}$$

$$Aerobic\ Glycolysis\ (Warburg\ effect): R_{o_2} = R_{Co_2} = 0, \qquad R_G = -\underbrace{k_m\,\gamma'_0 \upsilon}_{consumption} \tag{6}$$

γ_0 is the maximum consumption or production rate of the cellular respiration species in oxidative phosphorylation (OXPHOS) and γ'_0 is the maximum consumption rate of glucose in glycolysis; these are different for cancer and T cells (Table S1), and establishes competition for nutrients. Both cancer cells and effector T cells metabolize glucose via oxidative phosphorylation and glycolysis, with the Warburg effect introducing an additional glycolytic component [23-27]. For modeling purposes, in OXPHOS, we assume similar oxygen and glucose consumption rates for cancer cells and activated T cells. υ is the cellular vitality function defined in Eq. 20; ε_G, ε_{o_2}, and $\varepsilon_{co_2}$are natural decay rates of glucose, oxygen, and CO_2 into the interstitium. k_m is a constant defined as the intensity of cancer metabolism competition with T cells, used to control the metabolic rate of cancer cells.

1.1.2 tumor-induced paracrine signals for T cells:

Tumor-induced suppressive factors: the tumor produces suppressive factors (SFs), including Fas, on the ECs of tumor-vessels. We assume that the production rate of SFs is a constant, r_{sup}:

$$R_{sup} = \underbrace{r_{sup}\, C\ (X|X \in ECs\ \&\ CCs)}_{production\ by\ EC\ of\ tumor-vessels} - \underbrace{\varepsilon_{sup}\ c_{sup}}_{natural\ decay} \tag{7}$$

ε_{sup} is the natural decay of SFs.

Cytokines and Chemokines: cancer cells release cytokines and chemokines, respectively, to stimulate T cell adhesion to vessels and T cell migration within the intertium. We assume that cancer cells release two distinct types of cytokines: Type I cytokines, which promote rolling adhesion, and Type II cytokines, which facilitate firm adhesion. $r_{cyt1,2}$ and r_{chem} are poduction rates of type I and II cytokines and chemokines by cancer cells and $r'_{cyt1,2}$ and r'_{chem} are the production rates of cytokines and chemokines by cancer-T cell conjugates. r_{IL-2} is the poduction rate of IL-2 by CAR-T cells and r'_{IL-2} is the production rate of IL-2 by T-cancer cell conjugates. C and α refer to the identifications of cancer cells and cancer-T complexes weighted by cancer's status, including active populations (proliferating and migrating), quiescent cells, and necrotic cells.

In Eq. 8c, r'_{IL-2} is higher than r_{IL-2}. Indeed, when infiltrating CAR-T cells form conjugates with cancer cells, antigen-dependent activation occurs, leading to increased cytokine secretion that promotes CAR-T expansion within those regions. CAR-T cells produce cytokines at a very low basal rate; however, upon engagement with tumor cells, cytokine production increases substantially.

tumor cells can release more cytokines and chemokines when interacting with T cells. This is usually driven by inflammatory cytokines secreted by activated T cells. Therefore, in Eqs. 8a and 8b, r'_{cyt} and r'_{chem} are higher than r_{cyt} and r_{chem}.

$$R_{cyt1,2} = \underbrace{\left(r_{cyt1,2}\ C + r'_{cyt1,2}\ \alpha\right)}_{\substack{production\ by \\ cancer\ and\ cancer-T\ complex}} - \underbrace{\varepsilon_{cyt1,2}\ c_{cyt1,2}}_{natural\ decay} \tag{8a}$$

$$R_{chem} = \underbrace{\left(r_{chem}\ C + r'_{chem}\ \alpha\right)}_{\substack{production\ by \\ cancer\ and\ cancer-T\ complex}} - \underbrace{\varepsilon_{chem}\ c_{chem}}_{natural\ decay} \tag{8b}$$

Effector T cells also release IL-2, which is considered to control T cell proliferation of vital active T cells. r_{IL-2} is production rate of IL-2 with each effector T cell.

$$R_{IL-2} = \underbrace{\left(r_{IL-2}\ T + r'_{IL-2}\ \alpha\right)}_{\substack{production\ by\ eTs\ and \\ T-cancer\ complex}} - \underbrace{\varepsilon_{IL-2}\ c_{IL-2}}_{natural\ decay} \tag{8c}$$

1.1.3 vessel development and remodeling:

Because the vascular network of angiogenic vessels can be dynamically remodeled by proteins such as VEGF, Ang-1, and Ang-2—which regulate both branching and mechanical properties of vessel walls, particularly permeability—we incorporated these proteins and their receptor-mediated interactions into our model. By altering vascular structure at different tumor stages, these proteins can indirectly influence T cell trafficking. Moreover, VEGF can directly modulate lymphocyte adhesion mechanisms. Therefore, we account for the distribution of VEGF, Ang-1, and Ang-2, as well as their effects on T cell trafficking.

VEGF and VEGFR-2: the hypoxic TCs exposed to oxygen concentration below a threshold level, $c_{o_2}^{ch}$, secrete VEGF to stimulate ECs of nearby vessels to sprout. The VEGF also affects vascular hydraulic conductivity/pore size (Eq. 40). Once ECs become associated with the tumor tissue, their VEGFR-2 receptors are expressed [28]. Binding to and un-binding from VEGFR-2 act as a sink and source for VEGF, respectively. VEGF naturally decays in the interstitium. Therefore, the reaction model of VEGF coupled with VEGFR-2, oxygen, and ECM is governed by the set of equations 9 to 12:

$$R_v = \underbrace{r_{vegf}\left(1-\frac{c_{o_2}}{c_{o_2}^{ch}}\right)H_{(c_{o_2}^{ch}-c_{o_2})}}_{\substack{production by hypoxic\\ tumor cells}} - \underbrace{k_v^+ r_v^f c_v}_{\substack{bound\\ to VEGFR-2}} + \underbrace{k_v^- r_v^a}_{\substack{unbound\\ from VEGFR-2}} - \underbrace{\varepsilon_v c_v}_{natural decay} \tag{9}$$

$$R_v^b = \underbrace{k_v^+ r_v^f c_v}_{\substack{bound\\ to VEGFR-2}} - \underbrace{k_v^- r_v^a}_{\substack{unbound\\ from VEGFR-2}} \tag{10}$$

$$\frac{dr_v^f}{dt} = -k_v^+ r_v^f c_v + k_v^- r_v^a \tag{11}$$

$$\frac{dr_v^a}{dt} = k_v^+ r_v^f c_v - k_v^- r_v^a \tag{12}$$

R_v and R_v^b are the reaction rates of free VEGF and VEGF bound to VEGFR-2, respectively; c_v and c_v^b are concentrations of free and bound VEGF, respectively. r_{vegf} is the rate of production of VEGF by TCs; k_v^+ is the binding rate of VEGF to VEGFR-2; k_v^- is the dissociation rate of VEGF from VEGFR-2; k_{v-ag} is the VEGF binding rate to anti-VEGF; ε_v is the natural decay rate of VEGF; r_v^f is the concentration of free-VEGFR-2, and r_v^a is the concentration of active-VEGFR-2 bound to VEGF. As such, $H_{(c_{o_2}^{ch}-c_{o_2})}$ is a Heaviside function to activate VEFG secretion when oxygen concentration, c_{o_2}, falls below the characteristic value, $c_{o_2}^{ch}$.

ang-1 and ang-2 and their common receptor, Tie-2: ang-1 is secreted by ECs, and ang-2 is secreted by both ECs associated with tumor tissue and hypoxic tumor cells [28-30]. There is a competition between ang-1 and ang-2 to bind to their common Tie-2 receptor. Thus, competitive binding and unbinding of Tie-2 depletes or produces, respectively, ang-1 and ang-2. The reaction models of ang-1 and ang-2 coupled with Tie-2 also include natural decay, and are mathematically modeled through the set of equations 13 to19.

$$R_{a1} = \underbrace{r_{ang-1}\left(\frac{e_0 K_a-(c_{a1})^2}{K_a}\right)}_{production by endothelial cells} - \underbrace{k_{a1}^+ r_a^f c_{a1}}_{\substack{bound\\ to Tie-2}} + \underbrace{k_{a1}^- r_{a1}^a}_{\substack{unbound\\ from active Tie-2}} - \underbrace{\varepsilon_{a1} c_{a1}}_{natural decay} \tag{13}$$

$$R_{a1}^b = \underbrace{k_{a1}^+ r_a^f c_{a1}}_{\substack{bound\\ to Tie-2}} - \underbrace{k_{a1}^- r_{a1}^a}_{\substack{unbound\\ from active Tie-2}} \tag{14}$$

$$R_{a2} = r_{ang-2}^v\left(\frac{e_0 K_a-(c_{a2})^2}{K_a}\right) + r_{ang-2}^h\left(\frac{h_0 K_a-(c_{a2})^2}{K_a}\right) \tag{15}$$

$$- \underbrace{k_{a2}^{+} r_a^f c_{a2}}_{\substack{bound \\ toTie-2}} + \underbrace{k_{a2}^{-} r_{a2}^a}_{\substack{unbound \\ fromactiveTie-2}} - \underbrace{\varepsilon_{a2} c_{a2}}_{naturaldecay}$$

$$R_{a2}^b = \underbrace{k_{a2}^{+} r_a^f c_{a2}}_{\substack{bound \\ toTie-2}} - \underbrace{k_{a2}^{-} r_{a2}^a}_{\substack{unbound \\ fromactiveTie-2}} \tag{16}$$

$$\frac{dr_a^f}{dt} = -k_{a1}^{+} r_a^f c_{a1} + k_{a1}^{-} r_{a1}^a - k_{a2}^{+} r_a^f c_{a2} + k_{a2}^{-} r_{a2}^a \tag{17}$$

$$\frac{dr_{a1}^a}{dt} = k_{a1}^{+} r_a^f c_{a1} - k_{a1}^{-} r_{a1}^a \tag{18}$$

$$\frac{dr_{a2}^a}{dt} = k_{a2}^{+} r_a^f c_{a2} - k_{a2}^{-} r_{a2}^a \tag{19}$$

R_{a1},R_{a2}, R_{a1}^b, and $R_{a2}^b$are ,respectively, reaction rates of free ang-1 and ang-2, and bound ang-1 and ang-2 to Tie-2. c_{a1},c_{a2}, c_{a1}^b, and $c_{a2}^b$are, respectively, concentrations of free ang-1 and ang-2, and bound ang-1 and ang-2 to Tie-2. r_{ang-1}, r_{ang-2}^v, and r_{ang-2}^h are respectively the secretion rates of ang-1 by ECs, secretion rate of ang-2 by ECs associated with tumor tissue, and secretion rate of ang-2 by hypoxic TCs. $e_0$and h_0 are respectively the characteristic concentration of ECs in each blood vessel, and the characteristic concentration of TCs, $K_a$is the carrying capacity coefficient of angiopoietins,$k_{a1}^{+}$and$k_{a1}^{-}$are respectively ang-1 binding rate to and unbinding rate from Tie-2, k_{a2}^{+}andk_{a2}^{-} are respectively ang-2 binding rate to and unbinding rate from Tie-2,$\varepsilon_{a1}$and$\varepsilon_{a2}$are the natural decay rates of ang-1 and ang-2, respectively. r_a^f is the concentration of free Tie-2, $r_{a1}^a$and r_{a2}^a are concentrations of active Tie-2 bound to ang-1 and ang-2, respectively.

To consider fibronectin concentration in the ECM coupled with matrix metalloproteins (MMPs) secreted by ECs and TCs, we applied the reaction terms of our previous model [13].

1.2 Cellular Scale

1.2.1 Vitality of cancer and T cells

We implement a modified cellular vitality (υ)/cellular energy (ψ) model to consider the effects of oxygen, glucose and CO_2 on both cancer and T cell phenotypes [13]. Cellular vitality is increased with oxygen and glucose and decreased with CO_2. Cellular energy representing available units of ATP determine bioactivity of the cells [13,14,31]. The mathematical model of coupled cellular vitality and cellular energy is presented in Eqs. 20 and 21:

$$\upsilon = \varphi(\frac{c_{o_2}}{c_{o_2} + c_{o_2}^{ch}} + k_W).\frac{c_g}{c_g + c_g^{ch}} exp\left(-5(\frac{c_{co2}}{c_{co2}^{ch}} - 1)^4 H_{(c_{co_2} - c_{co_2}^{ch})}\right) \tag{20}$$

$$\frac{d\psi}{dt} = (k_a^p \upsilon - k_a^c \frac{\upsilon}{\upsilon+1} - k_{ac} c_{ac} \frac{\upsilon}{\upsilon+1}) H_{(\upsilon - \upsilon^{ch})} - k_q^c \frac{\upsilon}{\upsilon+1} H_{(\upsilon^{ch} - \upsilon)} \tag{21}$$

In Eq.20, φ is a proportionality coefficient, $c_{o_2}^{ch}$, c_g^{ch}, and c_{co2}^{ch} are oxygen, glucose, and carbon dioxide characteristic concentrations, respectively [13,32]. $H_{(c_{co_2}-c_{co_2}^{ch})}$is a Heaviside function to ensure that CO_2 reduces cellular vitality when it's concentration,c_{co_2}, exceeds the characteristic value,$c_{co_2}^{ch}$. k_W is a constant to reproduce the Warburg effect of cells, which tends to favor metabolism via glycolysis rather than the oxidative phosphorylation, which is the preference of most other cells in the body. Therefore, if the oxygen concentration of the cells approaches zero, the cells can survive but with very low vitality. Note, in the presence of oxygen, both cancer cells and T cells consume glucose through OXPHOS and glycolysis (Eq. 6). However, under anoxic conditions, they can still survive by relying solely on glycolysis.

In this model, the cells with υ below υ^{ch} are assumed to be quiescent and those with υ above $\upsilon^{ch}$are active [13]. The active cancer and T cells need to achieve a characteristic energy, ψ^{ch}, before they can proliferate into two new cells [13,33,34].

The active cells produce ATP at a linear rate related to cellular vitality with a proportional coefficient , k_a^p, and also consume cellular energy based on a M-M model with maximum rate k_a^c and M-M constant 1 [9,13]. The quiescent cells consume ATP according to a M-M model with maximum rate k_q^c and M-M constant 1. Quiescent cells with zero cellular energy are converted to necrotic phenotype. Indeed, quiescent cells can be converted to an active or necrotic state based on cellular vitality and energy, and active cells can become quiescent; however, necrotic cells cannot be converted to the other phenotypes.

1.2.2 Endothelial cells

During angiogenesis, endothelial tip cells (tECs) migrate toward positive gradients of VEGF [13,35-37] and stalk endothelial cells (sECs) migrate into the tECs-generated conduits in the ECM and also proliferate to create lumens of the angiogenic neo-vessels [13,35,38,39]. The death state is also considered for sECs based on VEGF concentration (see Eq.23). Moreover, the sECs can differentiate into tECs in response to high VEGF concentration and high ratio of ang-2 to ang-1, and thus generate bifurcating branches from the neo-vessel wall [35,40]. The branching probability function is presented in Eq. 22 [14].

$$P_{Br} = m_{Br} \frac{c_v^b}{c_v^b + k_{Br,v}} \frac{(c_{a2}^b/(c_{a1}^b + s_{Br,a}))}{(c_{a2}^b/(c_{a1}^b + s_{Br,a})) + k_{Br,a}} \tag{22}$$

m_{Br} is the maximum probability of branching, $k_{Br,v}$, and $k_{Br,a}$ are positive constants to control the impacts of VEGF and the ang-2 / ang-1 ratio on branching; $s_{Br,a}$ is a positive constant to avoid the singularity due to zero concentration of ang-1.

1.2.3 T cells and their interaction with ECs and cancer cells

Following CAR-T infusion, T cells circulate in the bloodstream, where they are captured through rolling adhesion, arrested by firm adhesion, and subsequently either extravasate or become suppressed (Fig. S1). After extravasation, they migrate within the interstitium via a random walk that is biased by haptotaxis and

chemotaxis in response to tumor-derived chemokines. Upon encountering cancer cells, CAR-T cells form complexes with them, which may result in dissociation, T cell exhaustion, or cancer cell killing.

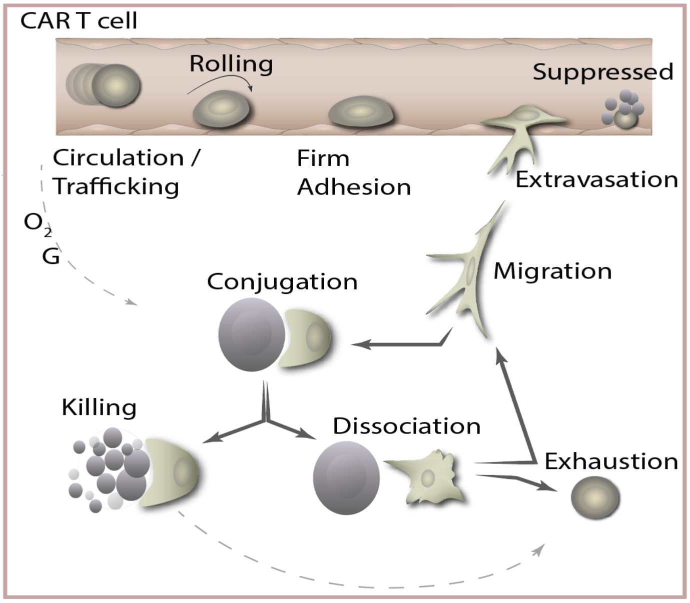

Figure S1. Schematics of T cell trafficking post-infusion interacting with ECs and tumor cells.

We start with free T cells flowing inside the angiogenic vessels, T_f; To create interstitial T cells, T_{ins}, the T_f cells contact the vessel wall and start rolling, T_r, with a defined rate on vessel walls, simulating selectin adhesions, and then switch to firmly adhered arrested T cells, T_{ad}, with defined a rate. Adhered cells can then transmigrate through ECs junctions to the interstitium.

Free T cells: We start with free T cells flowing inside the angiogenic vessels, T_f convected with blood flow in angiogenic vessels. We assume T cell release in the system is a Gaussian distribution with time with variance σ and peak-time t_p, simulating cell proliferation in lymph nodes and release into the circulation:

$$T_f = \frac{S_v}{\sqrt{2\pi\sigma^2}} \; e^{-(\frac{(t-t_p)^2}{2\sigma^2})} \tag{23}$$

$$S_v = S_T \; Q_{lum} \tag{24}$$

Where, S_T is T cell concentration (number of T cell per unit volume of blood), Q_{lum} flow rate of each vessel segment calculated by Eq. 37, then S_v is flux of T cells passing through the vessel segment. We assumed the CAR-T infusion dose is 10^7 cells and total volume of human blood is 5 lit, so $S_T = 2$ Mcell/lit.

Rolling adhered T cells; free T cells, T_f, are converted to rolling T cells, T_r, under the effect of Selectins, which we assume are activated by a type of cancer type I cytokines, c_{cyt1}. Each vessel segment is assumed

to have a limited capacity to capture rolling T cell, so we include a saturation term inversely related to the concentration of rolling T cells, T_r . these are modeled with the first term of Eq. 25. θ_{cyt1} is a M-M constant and β_{f-r} is rolling adhesion efficiency parameter. The second term of Eq.25 is the suppressive term, which simulates the elimination of rolling T cells by suppressive factors, c_{sup}, on the EC surface of vessels. k_{sup} is a M-M constant and ω_{sup} is the Suppressive factor strength parameter. The third term is the conversion of rolling T cells to firmly adhered T cells, which is affected by cancer type II cytokines, c_{cyt2}, which activate CAMs adhesion molecules on vascular endothelium. θ_{cyt2} is a M-M constant and β_{r-ad} is firm adhesion efficiency parameter. Factors such as VEGF, c_{vegf}, are considered to hinder the firm adhesion process. There is also a limited capacity to convert rolling T cell to adhered T cells considered by a saturation term. α_T is a constant that controls the saturation of T cell occupancy.

$$\frac{dT_r}{dt} = \underbrace{T_f \frac{c_{cyt1}}{c_{cyt1}+\theta_{cyt1}/\beta_{f-r}} \frac{1}{\alpha_T T_r + 1}}_{free\ to\ rolling\ phase} - \underbrace{T_r \frac{c_{sup}}{c_{sup}+k_{sup}/\omega_{sup}}}_{suppressive} - \underbrace{T_r \frac{1}{c_{vegf}+\theta_{vegf}} \frac{c_{cyt2}}{c_{cyt2}+\theta_{cyt2}/\beta_{r-ad}} \frac{1}{\alpha_T T_{ad} + 1}}_{rolling\ to\ adhesive\ phase} \quad (25)$$

Firmly Adhered T cells: the rolling T cells are converted to firm adhered T cells by CAMs activated by cancer type II cytokines, reduced by VEGF, and controlled by saturation capacity of vessel segment, respectively three parts of the first term. The second term is direct adhesion of free T cells. The third term is to calculate adhesion T cell converting rate to transmigrated T cells controlled by vessel wall permeability, L_p, and saturation capacity of interstitium vicinity of vessel, and the last term is calculating adhesion T cells suppression with suppressive factors,

$$\frac{dT_{ad}}{dt} = \underbrace{T_r \frac{1}{c_{vegf}+\theta_{vegf}} \frac{c_{cyt2}}{c_{cyt2}+\theta_{cyt2}/\beta_{r-ad}} \frac{1}{\alpha_T T_{ad} + 1}}_{rolling\ to\ adhesive\ phase} + \underbrace{T_f\, k_f}_{direct\ adhesive} - \underbrace{T_{ad}\, (\varepsilon + \frac{L_p}{L_p+L_p^0}) \frac{1}{\alpha_T T_{ad} + 1}}_{adhesive\ to\ transmigrative} - \underbrace{T_{ad} \frac{c_{sup}}{c_{sup}+k_{sup}/\omega_{sup}}}_{suppressive} \quad (26)$$

Extravasated T cells: Extravasated T cells migrate within the interstitium according to Eqs. (28a–c) and subsequently differentiate into complex (cytotoxic) T cells, T_{com}, after making complex with cancer cells with a spatiotemporal probability function, $\Theta(c)$, with rate k_1 and they can return after complex formation again with a spatiotemporal probability function, $\Theta(c)$ with rate k_{-1} . the cytotoxic T cell can also kill cancer and return to interstitium T cells but with a fractional rate, β .

$$\frac{dT_{ins}}{dt} = \underbrace{T_{ad}\, (\varepsilon + \frac{L_p}{L_p+L_p^0}) \frac{1}{\alpha_T T_{ad} + 1}}_{adhesive\ to\ transmigrative} + \underbrace{T_{com}\, k_{-1}}_{\substack{detachment\ from\\ intact\ cancer\ cells}} + \beta \underbrace{T_{com}\, \theta_1(T_{com}) k_2}_{\substack{detachment\ from\\ killed\ cancer\ cells}} - \underbrace{T_{ins}\, \Theta(c)\, k_1}_{ins\ to\ cytotoxic} \quad (27)$$

$$\Theta(c) = \begin{cases} c > 0 \begin{cases} r \le r_1 & 0 \\ r \ge r_1 & 1 \end{cases} \\ c = 0 \qquad\qquad 0 \end{cases}$$

In Eq. $\theta(c)$, $c > 0$ indicates that the T cell is in contact with a cancer cell. The parameter $r_1$represents the probability threshold for complex (conjugate) formation. A random number is generated for each T cell, r_1; if this value is greater than or equal to r_1, the T cell forms a conjugate with the cancer cell. If it is less than r_1, no complex is formed.

Migration of extravasated T cells: T cells chemotactically migrate towards positive gradients of chemokines, c_{chem}, with an intensity factor β_{ch}, and randomly walk with a diffusion coefficient D_t, their migration is also biased with ECM (fibronectin), c_e, gradient through haptotactic motion.

$$\frac{\partial T_{ins}}{\partial t} = D_t\, \nabla^2 T_{ins} - \nabla.(\beta_{ch}\, T_{ins}\, \nabla c_{chem}) - \nabla.(\beta_h\, T_{ins}\, \nabla c_e) \tag{28a}$$

$$\rho_{col} = A_{CAF}\, \sum_i e^{-\frac{{d_i}^2}{2\,\sigma^2}} \tag{28b}$$

$$P_T = \frac{P_0}{1+\beta_{col}\, \rho_{col}} \tag{28c}$$

T_{ins} represents the concentration of effector T cells in the interstitium; these cells can differentiate into cytotoxic T cells (T_{cyx}) or exhausted T cells (T_{exh}). T_{ins} cells migrate within the interstitium following a biased random walk. At locations occupied by cancer cells, collagen type I is produced, modeled using a Gaussian-like function as described in Eq. 28b. Although collagen is primarily produced by cancer-associated fibroblasts (CAFs), this function incorporates their effect implicitly. Here, σ is the spatial influence radius, set equal to the lattice length (50 µm), d is the Euclidean distance between neighboring cells and the central cell on the cancer lattice mesh, and $A_{CAF}$is the collagen production strength per CAF. Equation 28c calculates the probability of T cell migration to a new location, derived from Eq. 28a and modulated by the normalized collagen density. $P_0$is the baseline probability of migration in a collagen-free domain, set to 1, reflecting fast movement in the absence of physical barriers, and $\beta_{col}$is the collagen sensitivity factor for T cell migration in the ECM, set to 2.

CAR-T expansion: Following T cell recovery of vitality and energy, the T cells that are ready for proliferation expand in response to IL-2 according to Equation (28d).

$$\frac{dT_{ins}}{dt} = T_{ins}\, k_{IL-2}\, \frac{c_{IL-2}}{c_{IL-2}+\theta_{IL-2}}\, \frac{1}{\alpha_T\, T_{ins}+1} \tag{28d}$$

k_{IL-2} is maximum proliferation rate of CAR-T cells, and θ_{IL-2} is a M-M constant.

Once converted to cytotoxic T cells by successful binding to a cancer cell, they follow different dynamics.

Cytotoxic T cells; are made through complex formation with cancer cells, the first term, return to interstitium T cells, the second term, fractionally return to interstitium T cells, the third term, and exhausted by cancer cells, the last term, through PD-L/PD-1 mechanism modeled by spatiotemporal probability function of population of complex cytotoxic T cells with rate k_2.

$$\frac{dT_{com}}{dt} = \underbrace{T_{ins}\ \theta(c)\ k_1}_{ins\ to\ complex\ (cytotoxic)} - \underbrace{T_{com}\ k_{-1}}_{\substack{detachment\ from \\ intact\ cancer\ cells}} - \underbrace{T_{com}\ \theta_1(T_{com})\ k_2}_{\substack{detachment\ from \\ killed\ cancer\ cells}} - \underbrace{T_{com}\ \theta_2(T_{com})\ k_2}_{\substack{death\ before \\ killing\ cancer}} \tag{29}$$

Exhausted T cells; the cytotoxic T cells will be exhausted slightly after multiple cycles of killing cancer cells with probability θ_1, the first term, or immediately after complex with cancer with probability θ_2, the second term.

$$\frac{dT_{exh}}{dt} = \underbrace{(1-\beta)\ T_{com}\ \theta_1(T_{com})\ k_2}_{\substack{death\ after \\ killing\ cancer}} + \underbrace{T_{com}\ \theta_2(T_{com})\ k_2}_{\substack{death\ before \\ killing\ cancer}} \tag{30}$$

$$\theta_{1,2}(T_{com}) = \begin{cases} r' < r'_1 & \theta_1 = 0, \theta_2 = 1 \\ r' \geq r'_1\ and\ r' \leq r'_2 & \theta_1 = \theta_2 = 0 \\ r' \geq r'_2 & \theta_1 = 1, \theta_2 = 0 \end{cases} \tag{31}$$

In the $\theta_{1,2}(T_{com})$ equation, r_1'represents the threshold probability for exhaustion of conjugated T cells due to interaction with cancer cells, while r_2'denotes the threshold probability for cancer cell killing by the T cell. A random number $r'$is generated for each conjugated T cell to determine its fate during the cytotoxic phase: if $r' < r_1'$, the T cell becomes exhausted; if $r_1' \leq r' < r_2'$, the T cell remains in the conjugated state; and if $r' \geq r_2'$, the T cell successfully kills the cancer cell.

1.3 Tissue Scale

1.3.1 development of tumor tissue and neo-vessel pathways

In response to high VEGF concentration and VEGF gradients, tECs migrate into the ECM to create pathways for angiogenic neo-vessels. Cancer and T cells can sense the oxygen- and nutrient-rich regions in the tissue as well as cell density in the surrounding tissue. In addition to biochemical agents, tumor-induced solid pressure presents a resistance to the migration of TCs and tECs [9,13,41-43]. New TCs are stimulated to migrate toward the locations with high oxygen and nutrients (which can result in cooption of tumor vessels), low solid pressure (i.e., low viable cell concentration). We assume that newly-divided TCs can displace ("crowd") viable cells, but not necrotic cells. tECs are motivated to migrate toward high VEGF concentration regions and low solid pressure. The tECs cannot penetrate the regions occupied by necrotic TCs. The fibronectin gradient in the ECM caused by TC- and tEC-induced MMPs supports haptotactic migration of TCs and tECs [13].

$$\frac{\partial \rho_{tEC}}{\partial t} = \underbrace{D_{tEC} \nabla^2 \rho_{tEC}}_{Randomwalk} - \nabla . \left(\underbrace{\frac{\beta_c}{1+\alpha c_v} \rho_{tEC} \nabla c_v}_{chemotaxis} + \underbrace{\beta_h \rho_{tEC} \nabla c_e}_{Haptotaxis} \right) \tag{32}$$

$$\frac{\partial \rho_{CC}}{\partial t} = \underbrace{D_{TC} \nabla^2 \rho_{CC}}_{Randomwalk} - \nabla . \left(\underbrace{\beta_h \rho_{CC} \nabla c_e}_{Haptotaxis} + \underbrace{\beta_{cop} \rho_{CC} \nabla (\upsilon)}_{Cooption} \right) \tag{33}$$

$\rho_{tEC}$and $\rho_{CC}$are respectively tECs and cancer cells densities,D_{tEC} and $D_{TC}$are respectively diffusivity of tECs and cancer cells in the interstitium, α is a saturation coefficient for chemotaxis, β_c, β_h and β_{COP} are weight coefficients of chemotaxis, haptotaxis, and cooption, respectively. Other aspects of the tissue scale including vessel growth and remodeling, vessel deformation, and fluid dynamics of TME have been presented in our previous publication [14]. The parameters used for computational results of the mathematical model are listed in Table S1.

1.3.2 Vessel growth and remodeling

Lumenogenesis and vessel adaptation with growth factor and shear stress: After new vessels form via angiogenesis, they need to form lumens before flow can proceed. They do this through a process of lumenogenesis, which is controlled by VEGF, ang-1, and ang-2. The equation for neo-vessel diameter, d_v, is written as Eq. 34,

$$d_v = \frac{G_s}{G_s+G_0} d_c \tag{34}$$

$G_s$is the growth function of angiogenic neo-vessels and$G_0$is a M-M constant. $G_s$depends on the proliferation and death rates of sECs as well as their WSS-induced mechanotransduction, according to [44,45]:

$$\frac{dG_s}{dt} = \underbrace{\alpha_p \left(\frac{c_v^b}{c_v^b + \theta_p} \right) \underbrace{\left(1 - \delta_{ac} \frac{c_{ac}}{c_{ac} + \theta_{ac}} \right)}_{anti-cancer\ sEC\,\text{cytotoxicity}}}_{sEC\ proliferation} - \underbrace{\delta \left(1 - \frac{c_v^b}{c_v^b + \theta_d} \right)}_{sEC\ death} \\ + \underbrace{k_\tau (\tau_{WSS} - \tau_{WSS,ref}) H_{(c_v - c_{v,\min})} H_{(c_{v,\max} - c_v)}}_{Mechanotransduction-induced\ lumen\ growth} \tag{35}$$

α_p and θ_p are, respectively, the maximum rate and the M-M constant for sEC proliferation; $\delta_{ac}$and θ_{ac} are, respectively, the maximum rate and M-M constant for anti-cancer sEC cytotoxicity; $\delta$and $\theta_d$are the maximum rate and M-M constant for sEC death, respectively. $k_\tau$is also a positive constant that represents the neo-vessel lumen growth due to wall shear stress (WSS), $\tau_{WSS,ref}$ is a reference value for WSS, $c_{v,min}$ and $c_{v,max}$are threshold concentrations of VEGF, between which the WSS effect dominates.

Vessel deformation and adaptation with pressure: the angiogenic neo-vessels, which have a constant elasticity, E, and compliance power, cp, as well as a collapse pressure, p_c, are deformed through Eq. 36 [13,46-48].

$$d_{v,def} = d_v\left(\frac{p_{lum}-(p_{ins}+p_s)+p_c}{E}\right)^{cp} \quad (36)$$

p_{lum}, p_{ins}, and p_s are, respectively, intravascular pressure, IFP, and tumor growth-induced solid stress; d_v is vessel diameter and $d_{v,def}$ is deformed vessel diameter.

1.3.3 Fluid dynamics of the TME

Fluid transport is explicitly considered in the model. This includes the TME flow within vascular lumens, transvascular fluid flow, and interstitial fluid flow (IFF). Intravascular blood flow, governed by Hagen-Poiseuille's law, and IFF, calculated using Darcy's, law are coupled with each other through transvascular fluid flow calculated by Starling's law [6,11,13,49]. Equation 37 shows the continuity of intravascular blood flow. In this equation, Q_{lum} is the blood flow rate in the lumen calculated as the difference between intravascular blood flow rate, Q_{IBF}, based on Hagen-Poiseuille's law, Eq. 38, and transvascular fluid flow rate, Q_{TFF}, based on Starling's law, Eq. 39.

$$\sum_{b=1}^{N} Q_{lum}^{b}\,\beta^{b} = 0,\ Q_{lum} = Q_{IBF} - Q_{TFF} \quad (37)$$

$$Q_{IBF} = \frac{\pi}{128}\frac{\Delta p_{lum} {d_v}^4}{L\mu_{blood}(d_v, H_D)} \quad (38)$$

$$Q_{TFF} = (\pi d_v L)L_p(p_{lum} - p_{ins} - \sigma(\pi_{lum} - \pi_{ins})) \quad (39)$$

In Eq. 37, N is the number of peripheral vessel lattice nodes adjacent to the central vessel node, andβdescribes direction of lumen blood flow (+1 for outlet flow from a peripheral node and -1 for inlet flow to a peripheral node). In Eq. 38, L is the length of a neo-vessel segment, and$\mu_{blood}$is the dynamic non-Newtonian viscosity of blood as a function of neo-vessel diameter, d_v, and blood hematocrit, H_D, calculated using our previous hemorheology model [13]. In Eq. 39, $L_p$is the hydraulic conductivity of the neo-vessel wall, which is defined in Eq.40 as a function of VEGF and the ratio of ang-2 to ang-1 [47,50,51].

$$L_p = L_p^0\left(1 + k_p\frac{c_v^b}{c_v^b+k_{p,v}}\left(1 + \frac{(c_{a2}^b/c_{a1}^b)}{(c_{a2}^b/c_{a1}^b)+k_{p,a}}\right)\right) \quad (40)$$

$k_p$is a positive constant to limit the increment of L_p in response to VEGF and ang-2/ang-1, $k_{p,v}$and $k_{p,a}$are positive constants to control the effect of VEGF and ang-2/ang-1 on L_p, L_p^0 is a reference value for vessel wall hydraulic conductivity. The continuity equation for IFF given in Eq. 41 shows the incompressibility

of plasma in avascular tissue and leakiness of the neo-vessel wall in vascular tissue. Darcy's law, which determines IFF, is also shown in Eq. 42.

$$\nabla . u_{ins} = \begin{cases} \frac{Q_{TFF}}{V} = L_p S_V((p_{lum} - p_{ins} - \sigma(\pi_{lum} - \pi_{ins}))) & Vascular\ tissue \\ 0 & Avascular\ tissue \end{cases} \tag{41}$$

$$u_{ins} = -K_{ins} \nabla p_{ins} \tag{42}$$

By combining Eq.41 and Eq. 42, the Poison-Laplace's equation for IFP is derived (Eq. 43):

$$-\nabla^2 p_{ins} = \begin{cases} \frac{L_p}{K_{ins}} S_V((p_{lum} - p_{ins} - \sigma(\pi_{lum} - \pi_{ins}))) & Vascular\ tissue \\ 0 & Avascular\ tissue \end{cases} \tag{43}$$

$$K_{ins} = \begin{cases} K_{ins}^t \left(1 - k_{ps} \frac{p_s}{p_s + p_0}\right) & Tumor\ tissue \\ K_{ins}^s & Stroma\ tissue \end{cases} \tag{44}$$

$K_{ins}$is the interstitial hydraulic conductivity of the TME defined as a function of tumor-induced solid pressure in Eq.44, [6,7]. $K_{ins}^t$and $K_{ins}^s$are respectively the interstitial hydraulic conductivity of tumor and normal stroma tissue, k_{ps} is coefficient describing the reduction of hydraulic conductivity due to tumor fibrosis, and p_0 is a characteristic TME pressure.

Accumulation of rapidly dividing TCs increases the mechanical, compressive solid stress, p_s [9,52]. For this additional tumor growth-induced stress, we chose the Gaussian-like function for accumulative systems [9,13]. The model parameters are presented in Table S1.

Hotness factor: To assess how well the T cells distribute within the tumor tissue, we define a "hotness factor" (HF), calculated as Eq. 45, at each time point, *m* is the total number of living cancer cells, *n* the total number of spatial sites in the tumor that can be occupied by T cells (TCs), T_i the number of T cells in each site, and N_T the total number of T cells in the vicinity of cancer cell.

$$HF = \frac{\sum_{j=1}^{m} (\frac{\sum_{i=1}^{n} T_i N_T}{n})_j}{m} \tag{45}$$

2. Initial and boundary conditions

The initial concentrations of glucose, oxygen, and carbon-dioxide were assumed to be homogeneous. The initial concentrations of IL-2, cytokines, chemokines, SFs, VEGF, ang-1, and ang-2 are set to zero. The free and active VEGFR-2 and Tie-2 are initialized to zero. At the boundaries of the computational domain

of the TME, a Dirichlet boundary condition was used for each agent with value equal to its initial concentration. As in our previous model [13], the TME was seeded with five tumor cells located at the center of the computational cube and a hypothetical circular primary vascular network with a radius approximately 5 mm. The locations of initial sprouts on the circle of primary vessels are determined based on VEGF concentration but spaced randomly according to NOTCH induction more than 50 µm apart.

The biomechanical factors, including IFF velocity and IFP, intravascular blood flow velocity and pressure, and WSS were initially set to zero in the entire computational domain and boundaries. For these parameters, a Dirichlet boundary condition with zero value was set on all boundaries of the TME domain. To allow for increased blood supply and vascular maturation with tumor growth, we assume the surrounding supply vessels grow and their pressure increase as the tumor grows: at the inlet of the neo-vessels where they connect to the primary vessel, we developed a M-M model to calculate inlet pressure as a function of tumor size; $p_{inlet} = p_m(V_T/(V_T^m + V_T))$, where V_T is tumor volume, V_T^m is M-M constant, and p_m is the maximum pressure in the primary vessel, consistent with the range reported in the literature [7,10,53].

3. Assumptions and limitations

A) the primary vessels are assumed to be on a circular region. B) concentrations in the far-field are assumed to be constant. C) The dynamics of stromal cells are not considered.

4. T cell distribution in the TME:

We spatially validated our model by comparing T cell distributions across normal tissue and vessels, peritumoral vessels, and intratumoral abnormal vessels—patterns commonly observed in vascularized solid tumors[54-57](Fig. S2). These characteristic perivascular, infiltrative, and intratumoral distributions of T cells have been widely reported across many solid tumor types. The spatiotemporal distribution of T cells in our simulated tumor TME shows that most T cells accumulate around the tumor periphery, while fewer cells extravasate into the tumor core. This finding suggests that the conventional "cold" versus "hot" tumor classification may be oversimplified: a "cold" tumor (with few or peripherally localized T cells and a low hotness factor, HF) can potentially be converted into a "hot" tumor through modulation of T cell–vessel–tumor interactions (see Fig. 4 in the main text).

We further performed both qualitative and quantitative validations by comparing our baseline physiological tumor model with the experimental findings of Schoenberg, et al. [58] (Fig. S3). Our simulations reproduced similar perivascular T cell localization patterns within the tumor as observed experimentally in non-vascular tumor regions. This accumulation of T cells near tumor vessels and around the tumor mass, driven by physical and structural barriers, is a well-recognized hallmark of solid tumors responding to immunogenic T cells or systemically delivered CAR-T cells [58,59].

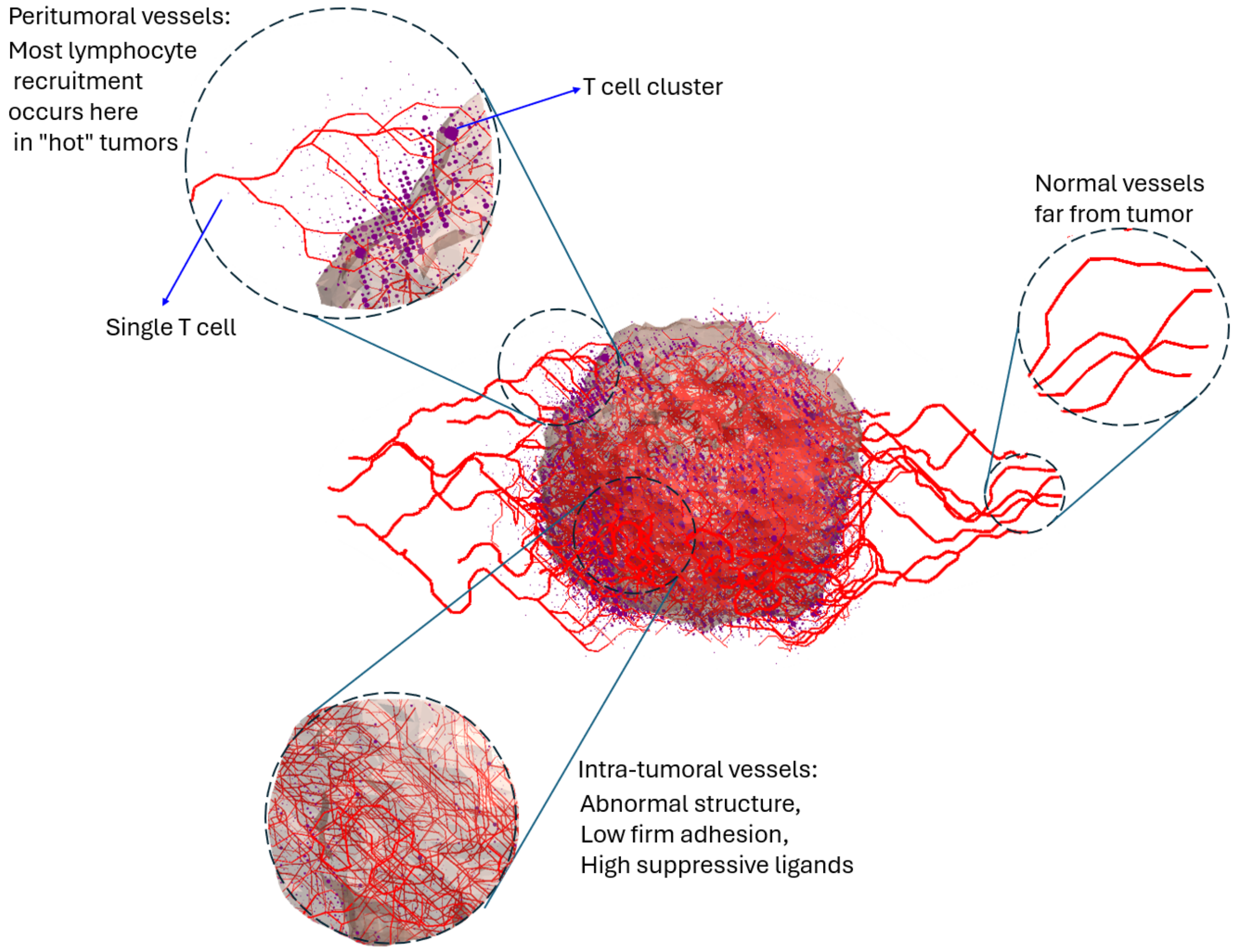

Figure S2. T cell distribution in the TME. Shown are T cell distributions across three distinct zones of the TME—normal vessels distant from the tumor, peritumoral vessels, and intratumoral vessels—illustrating how tumor–vessel interactions influence the efficiency of T cell infiltration following systemic CAR-T infusion.

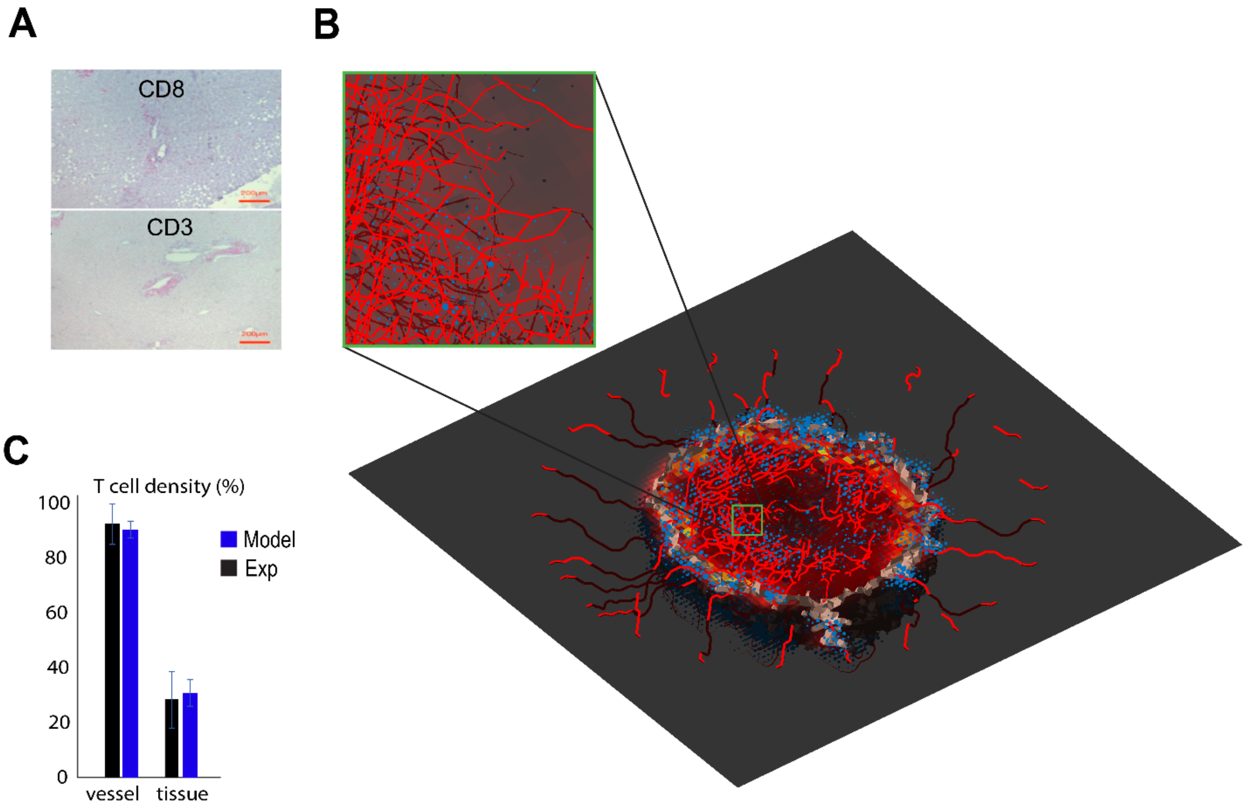

Figure S3. T cell distribution within the tumor. The spatial distribution of T cells in perivascular and non-vascular intratumoral regions shows good agreement with the experimental results reported by Schoenberg, et al. [58]. Panel A shows representative perivascular patterns of $CD3^{+}$ and $CD8^{+}$ T cells from tumors of 60 patients reported by Schoenberg, et al. [58]. Panel B presents our spatiotemporal simulation of a baseline tumor, demonstrating T cell distribution in perivascular versus non-vascular regions with qualitatively similar trends to experimental observations. Panel C compares the quantitative T cell densities in perivascular and non-vascular tumor regions between the experimental data and our computational results.

Correlation between HF and tumor regression:

In Figure S4, we show the correlation between HF and tumor regression (measured as tumor size reduction ratio) across wide parameter sweeps of the six calibrated parameters. Based on the slopes of these correlation plots and the range of HF variations for each parameter (Fig. S4), our analysis indicates that the model's key conclusions are primarily influenced by collagen density and metabolic competition, followed by tumor-vessel suppressive factor, firm adhesion, and rolling adhesion. Variations in chemotactic strength have negligible impact, confirming that the main findings regarding CAR-T infiltration and cytotoxic efficacy are robust to uncertainty in less-constrained parameters.

To assess robustness within the calibrated parameter ranges, posterior samples obtained from the Bayesian calibration are overlaid in Figure 4 (shown as *). HF and tumor regression trends remain consistent across these posterior samples. Positive correlations are maintained for collagen, adhesion, and suppressive signaling, while metabolic competition consistently shows negative correlation, and chemotactic strength

has minimal effect. These observations indicate that HF is robust to parameter uncertainty and mechanistically interpretable.

HF is mechanistically interpretable because it reflects the combined contributions of well-characterized biological processes: T-cell rolling and firm adhesion (intravascular trafficking), suppressive endothelial signaling, ECM density, metabolic competition, and chemotactic migration. Posterior analysis confirms that changes in HF correspond to the expected mechanistic effects of these parameters. In particular, the negative correlation between HF and tumor regression for metabolic competition reflects underlying biology: high metabolic competition reduces T-cell activity (lower HF) but simultaneously limits tumor cell growth via nutrient depletion. As a result, tumor regression can still occur even when HF is low.

Together, these findings demonstrate that HF provides a direct link to underlying biological mechanisms rather than representing a black-box metric, and that the mechanistic conclusions derived from HF are robust across the posterior distribution.

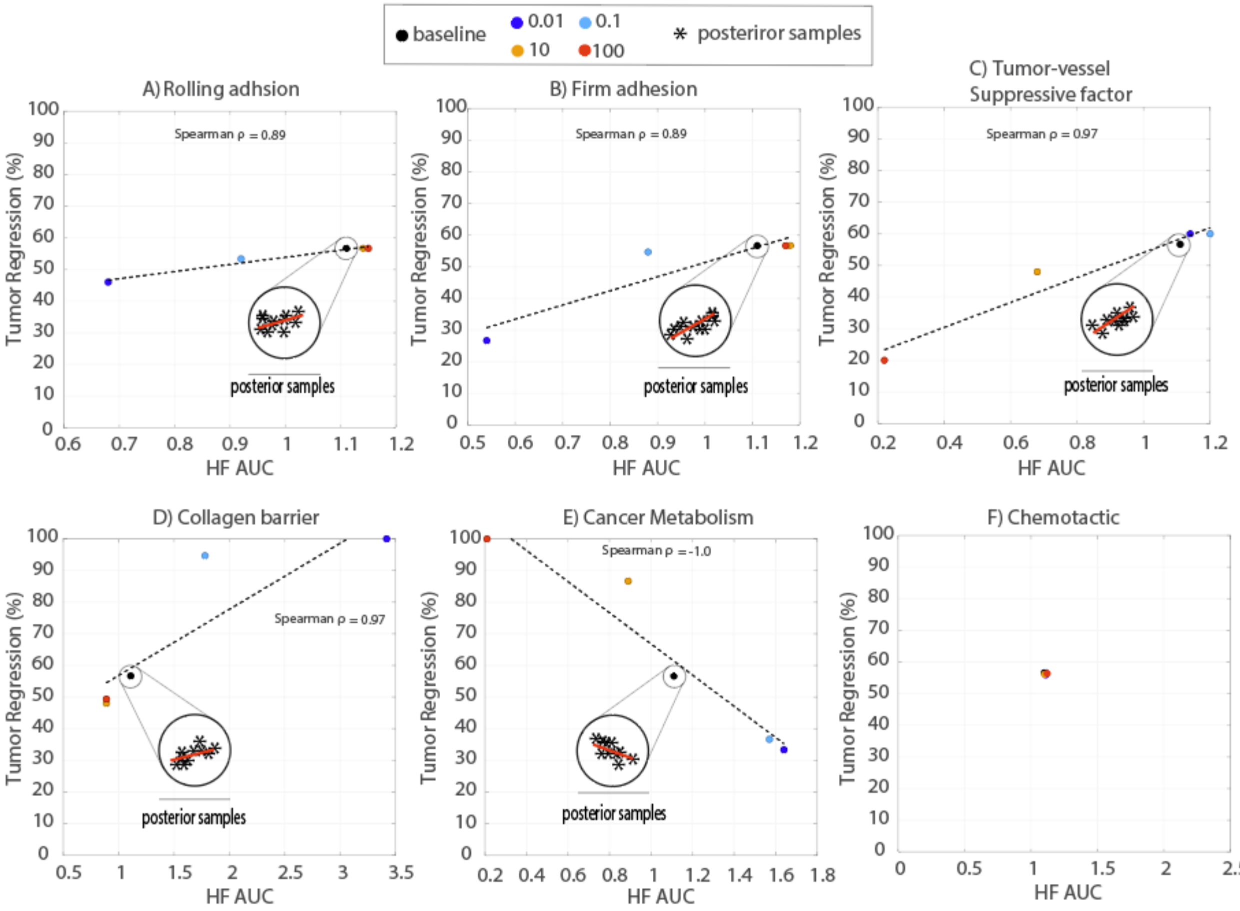

Figure S4. Correlation between HF and tumor regression and robustness of HF to parameter uncertainty.

Table S1. The parameters used for computational results of the mathematical model.

Parameters	Description	Value	References
Cellular respiration			
D_{o_2}	Diffusion coefficient of oxygen	1×10^{-9} m^2/s	Anderson [60]
D_G	Diffusion coefficient of glucose	3.6×10^{-10} m^2/s	Jain [61]
D_{co_2}	Diffusion coefficient of carbon-dioxide	7.4×10^{-10} m^2/s	Laursen and Kirk [62]
γ_0	Maximum consumption/production rate of CR-agents	3×10^{-8} M/s	Tang, et al. [9]
γ'_0	Maximum consumption rate of glucose (glycolysis)	Cancer: 1.2×10^{-7} M/s<br>T cell : 0.3×10^{-7} M/s	[23-27]
k_{met}	cancer metabolism intensity	1	Estimated (for sensitivity analysis)
$c_{o_2}^{ch}$	Characteristic oxygen concentration (threshold value for hypoxia)	8.4×10^{-3} M	Tang, et al. [9]
c_G^{ch}	Characteristic glucose concentration (threshold value for hypoglycemia)	5.5×10^{-3} M	Bao, et al. [63]
$c_{co_2}^{ch}$	Characteristic carbon-dioxide concentration	10.5×10^{-3} M	Tang, et al. [9]
c_{p,o_2}	plasma concentration of oxygen	8.4×10^{-3} M	Tang, et al. [9]
$c_{p,G}$	plasma concentration of glucose	5.5×10^{-3} M	Bao, et al. [63]
c_{p,co_2}	plasma concentration of CO2	1.2×10^{-3} M	Higgins [64]
ε_{o2}	Natural decay rate of oxygen	1×10^{-9} 1/s	Estimated
ε_G	Natural decay rate of glucose	1.7×10^{-10} 1/s	Estimated
ε_{co2}	Natural decay rate of carbon-dioxide	1×10^{-10} 1/s	Estimated
r_f	retardation factor	1	Baxter and Jain [18]
T cell interaction molecules			
D_{IL-2}	Diffusion coefficient of IL-2	3×10^{-11} m^2/s	Anderson and Chaplain [65,66,67]
D_{chem}	Diffusion coefficient of chemokines	1×10^{-11} m^2/s	[68]
D_{cyt}	Diffusion coefficient of cytokines	1×10^{-11} m^2/s	[69]
D_{SF}	Diffusion coefficient of SF	0.5×10^{-11} m^2/s	[70]
r_{IL-2}	IL-2 production rate by T cells ($r'_{IL-2} = 10\, r_{IL-2}$)	1×10^{-10} M/s	[71]
r_{chem}	Chemokine production rate by Cancer cells ($r'_{chem} = 10\, r_{chem}$)	2×10^{-11} M/s	[72]
$r_{cyt1,2}$	cytokine production rate by Cancer cells ($r'_{cyt1,2} = 10\, r_{cyt1,2}$)	1×10^{-11} M/s	[73]
r_{SF}	SF production rate by Cancer cells	1×10^{-11} M/s	[74]
ε_{IL-2}	Natural decay rate of IL-2	5.8×10^{-4} 1/s	[75,76]
ε_{chem}	Natural decay rate of chemokines	3.9×10^{-4} 1/s	Estimated
ε_{cyt}	Natural decay rate of type I and II cytokines	3.9×10^{-4} 1/s	Estimated
ε_{SF}	Natural decay rate of SF	6.4×10^{-5} 1/s	Estimated
θ_{cyt1}	M-M half-saturation constant for free to rolling T cells	2 ng/ml	Estimated
θ_{cyt2}	M-M half-saturation constant for rolling to firm adhered T cells	5 ng/ml	Estimated
k_{sup}	M-M half-saturation constant for suppressed T cells	1 ng/ml	Estimated
k_{IL-2}	maximum proliferation rate constant	1.5 1/day	[77] Estimated
θ_{IL-2}	M-M half-saturation constant for proliferation of T cells with IL-2	1 ng/ml	[77] Estimated
Growth factors			
D_v	Diffusion coefficient of VEGF	2.9×10^{-11} m^2/s	Anderson and Chaplain [65,66]
r_{vegf}	VEGF production rate by Cancer cells	2×10^{-12} M/s	Tang, et al. [9]
k_v^+	Binding rate of VEGF by VEGFR-2	1.3×10^{-2} 1/(μM.s)	Baldwin, et al. [78]
k_v^-	Unbinding rate of VEGF from VEGFR-2	6.3×10^{-5} 1/s	Baldwin, et al. [78]
ε_v	Natural decay rate of VEGF	2.78×10^{-7} 1/s	Gevertz and Torquato [28]
c_v^{ch}	Characteristic VEGF concentration	1.1×10^{-8} M	Tang, et al. [9]
$c_{p,v}$	plasma concentration of VEGF	5.78 pg/ml	McIlhenny, et al. [79]
Angiopoietins			
D_{a1}	Diffusion coefficient of ang-1	Very small →0 m^2/s	Gevertz and Torquato [28]
D_{a2}	Diffusion coefficient of ang-2	12.5×10^{-13} m^2/s	Billy, et al. [80]
r_{ang-1}	Secretion rate of ang-1 by ECs	2.78×10^{-6} 1/s	Gevertz and Torquato [28]
r_{ang-2}^v	Secretion rate of ang-2 by ECs associated with tumor tissue	2.22×10^{-5} 1/s	Gevertz and Torquato [28]
r_{ang-2}^h	secretion rate of ang-2 by Cancer cells	1.4×10^{-5} 1/s	Gevertz and Torquato [28]

Parameters	Description	Value	References
k_{a1}^{+}	Binding rate of ang-1 to Tie-2	1×10^{-2} 1/(μM.s)	Davis, et al. [81]<br>Longstaff [82]
k_{a1}^{-}	Unbinding rate of ang-1 from Tie-2	3.7×10^{-5} 1/s	Davis, et al. [81]<br>Longstaff [82]
k_{a2}^{+}	Binding rate of ang-2 to Tie-2	1.16×10^{-2} 1/(μM.s)	Maisonpierre, et al. [83]<br>Gevertz and Torquato [28]
k_{a2}^{-}	Unbinding rate of ang-2 from Tie-2	3×10^{-5} 1/s	Maisonpierre, et al. [83]<br>Gevertz and Torquato [28]
K_a	Carrying capacity coefficient of angiopoietins	1.5×10^{-2} μM	Gevertz and Torquato [28]
e_0	Characteristic concentration of ECs at each blood vessel	1×10^{-4} μM	Plank, et al. [84]
h_0	Characteristic concentration of TCs	1×10^{-3} μM	Gevertz and Torquato [28]
ε_{a1}	Natural decay rates of ang-1	8.33×10^{-7} 1/s	Gevertz and Torquato [28]
ε_{a2}	Natural decay rates of ang-2	5.56×10^{-7} 1/s	Gevertz and Torquato [28]
$c_{p,a1}$	plasma concentration of ang-1	6×10^{3} pg/ml	Engin, et al. [85]
$c_{p,a2}$	plasma concentration of ang-2	2×10^{3} pg/ml	Engin, et al. [85]
**Extracellular matrix**			
D_m	Diffusion coefficient of MMPs	1×10^{-13} m^2/s	Cai, et al. [86]
$r_{m,T}$	Secretion rates of MMPs by TCs	1.7×10^{-13} M/s	Nikmaneshi, et al. [13]
$r_{m,E}$	Secretion rates of MMPs by ECs	0.3×10^{-13} M/s	Nikmaneshi, et al. [13]
ε_m	Natural decay rates of MMPs	1.7×10^{-8} 1/s	Cai, et al. [86]
ε_e	Natural decay rates of ECM	1.3×10^{-7} 1/s	Cai, et al. [86]
c_e^{ch}	Characteristic ECM concentration	1.36×10^{-9} M	Nikmaneshi, et al. [13]
c_m^{ch}	Characteristic MMP concentration	1.36×10^{-9} M	Nikmaneshi, et al. [13]
$c_{p,m}$	plasma concentration of MMP	72 ng/ml	Masuhara, et al. [87]
**Cellular vitality and energy**			
φ	Proportionality coefficient of consumption/production rate of CR-agents	3.67	Buchwald [32]
υ^{ch}	Characteristic cellular vitality for active TCs	0.5	Nikmaneshi, et al. [13]
ψ^{ch}	Characteristic cellular energy for proliferation	30	Nikmaneshi, et al. [13]
k_q^c	Constant consumption rate of cellular energy by quiescent TCs	0.1	Tang, et al. [9] Nikmaneshi, et al. [13]
k_a^p	Coefficient of production rate of cellular energy by active TCs	1	Nikmaneshi, et al. [13]
k_a^c	Maximum consumption rate of cellular energy by active TCs	1	Nikmaneshi, et al. [13]
k_W	a constant to demonstrate the Warburg effect of TCs	1×10^{-10}	Estimated
**Tumor growth and angiogenesis**			
α	1	Saturation coefficient of chemotaxis	Cai, et al. [86]
β_c	0.26 m^2/(M.s)	Weight coefficient of chemotaxis	Cai, et al. [86]
β_h	0.1 m^2/(M.s)	Weight coefficient of haptotaxis	Cai, et al. [86]
β_{COP}	0.3 m^2/(M.s)	Weight coefficient of Cooption	Estimated
D_{tEC}	Diffusivity of tECs	1×10^{-13} m^2/s	Cai, et al. [86]
D_{TC}	Diffusivity of TCs	1×10^{-13} m^2/s	Cai, et al. [86]
**Vessel growth and remodeling**			
G_0	M-M constant of neo-vessel lumen growth	500	Nikmaneshi, et al. [13]
α_p	Maximum rate of sEC proliferation	0.198 1/h	Cameron and Davis [44]
θ_p	M-M constant of sEC proliferation	2.8×10^{-6} mol VEGF/ 1 m^3 EC	Cameron and Davis [44]
δ	Maximum rate of sECs death	0.198 1/h	Cameron and Davis [44]
δ_{ac}	maximum rate for anti-cancer sECs cytotoxicity	0.67 1/h	Estimated, Woodley-Cook, et al. [88]
θ_{ac}	M-M constant for anti-cancer sECs cytotoxicity	5.2 μg/ml	Estimated, Woodley-Cook, et al. [88]
θ_d	M-M constant of sECs death	3.3×10^{-7} mol VEGF/ 1 m^3 EC	Cameron and Davis [44]
$\tau_{WSS,ref}$	A positive constant as reference of WSS	7.73×10^{-5} mmHg	Pries, et al. [89]
$c_{v,min}$	Minimum threshold concentrations of VEGF, above which the WSS effect can be dominant	1.1×10^{-11} M	Estimated
$c_{v,max}$	Maximum threshold concentrations of VEGF, below which the WSS effect can be dominant	1.1×10^{-5} M	Estimated

Parameters	Description	Value	References
k_p	Proportional coefficient of transvascular stimuli	0.5 1/s	Stéphanou, et al. [90]
k_m	Proportional coefficient of metabolic stimuli	0.12 1/s	Stéphanou, et al. [90]
S_{Sh}	Inherent tendency of vessels to shrink and decrease their diameter	0.35	Stéphanou, et al. [90]
E	Constant elasticity of neo-vessels	6.5 mmHg	Netti, et al. [46]
cp	Compliance power of neo-vessels	0.19	Netti, et al. [46]
p_c	Collapse pressure of neo-vessels	3 mmHg	Netti, et al. [46]
d_c	Characteristic diameter of neo-vessels	50 µm	Nikmaneshi, et al. [13]
m_{Br}	Branching constant	0.3×10^{-3}	Tang, et al. [9]
$k_{Br,v}, k_{Br,a}$	positive constants to control the impact of ratio of ang-2 to ang-1 on branching	1	Estimated
Hemodynamics-interstitial fluid flow of TME			
$K^s{}_{ins}$	Interstitial hydraulic conductivity of TME for normal tissue	8.53×10^{-9} cm2/(mmHg.s)	Baxter and Jain [18]
$K^t{}_{ins}$	Interstitial hydraulic conductivity of TME for tumor tissue	4.13×10^{-8} cm2/(mmHg.s)	Baxter and Jain [18]
k_{ps}	tumor-induced reduction coefficient of hydraulic conductivity	1	Estimated
L_p^0	Reference value for Hydraulic conductivity of angiogenic neo-vessels wall	3.6×10^{-10} m/(mmHg.s)	Cai, et al. [47], Cai, et al. [51]
k_L	constant to control the effect of VEGF and ang-2/ang-1 on vessel wall hydraulic conductivity	3.39	Baxter and Jain [18]
$k_{L,v}$	constant to control the effect of VEGF on vessel wall hydraulic conductivity	1.1×10^{-8} M (equal to characteristic value of VEGF)	Estimated
$k_{L,a}$	constant to control the effect of ang-2/ang-1 on vessel wall hydraulic conductivity	1	Estimated
σ_v	average oncotic reflection coefficient of plasma proteins	0.91 for healthy tissue and 0.82 for tumor tissue	Soltani and Chen [6], Zhao, et al. [7]
π_{lum}	collide osmotic (oncotic) pressures of intravascular plasma	20 mmHg for both healthy and tumor tissues	Soltani and Chen [6], Zhao, et al. [7]
π_{ins}	collide osmotic (oncotic) pressures of interstitial fluid	10 mmHg for heathy tissue and 15 mmHg for tumor tissue	Soltani and Chen [6], Zhao, et al. [7]
S_V	Characteristic value of surface area of neo-vessels per unit volume for mass transport in the interstitium	7×10^3m^{-1} for healthy tissue and 2×10^4m^{-1} for tumor tissue	Soltani and Chen [6], Zhao, et al. [7]
p_0	Characteristic TME pressure and WSS	60 mmHg	Tang, et al. [9]
p_m	the maximum pressure in the primary vessels	60 mmHg	[7,10,53]
d_p^t	Intratumoral vessel wall pore size	400 nm	Stylianopoulos and Jain [91]
κ_i	Permeability coefficient of vessel wall	5.73×10^{-9} cm/s for tumor tissue<br>0.73×10^{-9} cm/s for normal tissue	Baxter and Jain [19]

Bayesian Posterior Analysis of Calibrated CAR-T Parameters:

Rolling and firm adhesion efficiencies exhibit strong and very good practical identifiability, respectively, with a mild positive correlation reflecting their sequential roles in T-cell extravasation. Suppressive signaling, collagen, metabolic competition, and chemotactic parameters remain narrow and largely independent, confirming the model's practical identifiability and mechanistic separation of intravascular and extravascular processes.

Table S2. Posterior summary of the six calibrated CAR-T trafficking parameters, including medians, 95% credible intervals, coefficient of variation, pairwise correlations, and practical identifiability.

Parameter	Biological Role	Posterior Median	95% Credible Interval	CV (%)	Max Pairwise Correlation (ρ)	Practical Identifiability
Rolling Adhesion Efficiency (β_{f-r})	T-cell rolling along endothelium	0.62	[0.55 – 0.69]	11%	0.42 (with Firm Adhesion)	Strong
Firm Adhesion Efficiency (β_{r-ad})	Stable endothelial attachment	0.71	[0.65 – 0.77]	9%	0.42 (with Rolling)	Very Good
Suppressive Factor Strength (ω_{sup})	Endothelial inhibitory modulation	0.54	[0.49 – 0.60]	7%	< 0.10	Strong
Collagen production strength (A_{CAF})	Physical ECM barrier	0.68	[0.61 – 0.74]	6%	< 0.10	Strong
Metabolic Competition Intensity (k_{met})	Nutrient competition	0.59	[0.53 – 0.65]	8%	< 0.15	Strong
Chemotactic Strength (β_{ch})	Directed T-cell chemotactic migration	0.73	[0.67 – 0.80]	7%	< 0.10	Strong

References:

1 Yonucu, S., Yılmaz, D., Phipps, C., Unlu, M. B. & Kohandel, M. Quantifying the effects of antiangiogenic and chemotherapy drug combinations on drug delivery and treatment efficacy. *PLoS computational biology* **13**, e1005724 (2017).

2 Jain, R. K., Tong, R. T. & Munn, L. L. Effect of vascular normalization by antiangiogenic therapy on interstitial hypertension, peritumor edema, and lymphatic metastasis: insights from a mathematical model. *Cancer research* **67**, 2729–2735 (2007).

3 Xu, J., Vilanova, G. & Gomez, H. A mathematical model coupling tumor growth and angiogenesis. *PloS one* **11**, e0149422 (2016).

4 Voutouri, C. *et al.* Experimental and computational analyses reveal dynamics of tumor vessel cooption and optimal treatment strategies. *Proceedings of the National Academy of Sciences* **116**, 2662–2671 (2019).

5 Kashkooli, F. M., Soltani, M., Rezaeian, M., Taatizadeh, E. & Hamedi, M.-H. Image-based spatio-temporal model of drug delivery in a heterogeneous vasculature of a solid tumor—Computational approach. *Microvascular research* **123**, 111–124 (2019).

6 Soltani, M. & Chen, P. Numerical modeling of interstitial fluid flow coupled with blood flow through a remodeled solid tumor microvascular network. *PloS one* **8**, e67025 (2013).

7 Zhao, G. *et al.* Numerical simulation of blood flow and interstitial fluid pressure in solid tumor microcirculation based on tumor-induced angiogenesis. *Acta Mechanica Sinica* **23**, 477–483 (2007).

8 Norton, K.-A. & Popel, A. S. Effects of endothelial cell proliferation and migration rates in a computational model of sprouting angiogenesis. *Scientific reports* **6**, 36992 (2016).

9 Tang, L. *et al.* Computational modeling of 3D tumor growth and angiogenesis for chemotherapy evaluation. *PloS one* **9**, e83962 (2014).

10 Stéphanou, A. *et al.* How tumour-induced vascular changes alter angiogenesis: Insights from a computational model. *Journal of theoretical biology* **419**, 211–226 (2017).

11 Vavourakis, V. *et al.* A validated multiscale in-silico model for mechano-sensitive tumour angiogenesis and growth. *PLoS computational biology* **13**, e1005259 (2017).

12 Shamsi, M., Saghafian, M., Dejam, M. & Sanati-Nezhad, A. Mathematical modeling of the function of Warburg effect in tumor microenvironment. *Scientific reports* **8**, 1–13 (2018).

13 Nikmaneshi, M. R., Firoozabadi, B., Mozafari, A. & Munn, L. L. A multi-scale model for determining the effects of pathophysiology and metabolic disorders on tumor growth. *Scientific reports* **10**, 1–20 (2020).

14 Nikmaneshi, M. R., Jain, R. K. & Munn, L. L. Computational simulations of tumor growth and treatment response: benefits of high-frequency, low-dose drug regimens and concurrent vascular normalization. *PLoS Computational Biology* **19**, e1011131 (2023).

15 Nikmaneshi, M. R., Firoozabadi, B., Mozafari, A. & Munn, L. L. A multi-scale model for determining the effects of pathophysiology and metabolic disorders on tumor growth. *Scientific reports* **10**, 3025 (2020).

16 Nikmaneshi, M. R., Firoozabadi, B. & Mozafari, A. Chemo-mechanistic multi-scale model of a three-dimensional tumor microenvironment to quantify the chemotherapy response of cancer. *Biotechnology and Bioengineering* **118**, 3871–3887 (2021).

17 Nikmaneshi, M. R. & Firoozabadi, B. Investigation of cancer response to chemotherapy: a hybrid multi-scale mathematical and computational model of the tumor microenvironment. *Biomechanics and Modeling in Mechanobiology* **21**, 1233–1249 (2022).

18 Baxter, L. T. & Jain, R. K. Transport of fluid and macromolecules in tumors. I. Role of interstitial pressure and convection. *Microvascular research* **37**, 77–104 (1989).

19 Baxter, L. T. & Jain, R. K. Transport of fluid and macromolecules in tumors. II. Role of heterogeneous perfusion and lymphatics. *Microvascular research* **40**, 246–263 (1990).

20 Baxter, L. T. & Jain, R. K. Transport of fluid and macromolecules in tumors: III. Role of binding and metabolism. *Microvascular research* **41**, 5–23 (1991).

21 Baxter, L. T. & Jain, R. K. Transport of fluid and macromolecules in tumors. IV. A microscopic model of the perivascular distribution. *Microvascular research* **41**, 252–272 (1991).

22 Jain, R. K. Vascular and interstitial barriers to delivery of therapeutic agents in tumors. *Cancer and Metastasis Reviews* **9**, 253–266 (1990).

23 Zheng, J. Energy metabolism of cancer: Glycolysis versus oxidative phosphorylation. *Oncology letters* **4**, 1151–1157 (2012).

24 Tufail, M., Jiang, C.-H. & Li, N. Altered metabolism in cancer: insights into energy pathways and therapeutic targets. *Molecular cancer* **23**, 203 (2024).

25 Klein Geltink, R. I., Kyle, R. L. & Pearce, E. L. Unraveling the complex interplay between T cell metabolism and function. *Annual review of immunology* **36**, 461–488 (2018).

26 Shi, Y., Zhang, H. & Miao, C. Metabolic reprogram and T cell differentiation in inflammation: current evidence and future perspectives. *Cell Death Discovery* **11**, 123 (2025).

27 Cao, J. *et al.* Effects of altered glycolysis levels on CD8+ T cell activation and function. *Cell death & disease* **14**, 407 (2023).

28 Gevertz, J. L. & Torquato, S. Modeling the effects of vasculature evolution on early brain tumor growth. *Journal of Theoretical Biology* **243**, 517–531 (2006).

29 Baffert, F. *et al.* Age-related changes in vascular endothelial growth factor dependency and angiopoietin-1-induced plasticity of adult blood vessels. *Circulation research* **94**, 984–992 (2004).

30 Carmeliet, P. Angiogenesis in health and disease. *Nature medicine* **9**, 653 (2003).

31 Berk, A., Zipursky, S. & Lodish, H. (National Center for Biotechnology InformationÕs Bookshelf, 2000).

32 Buchwald, P. FEM-based oxygen consumption and cell viability models for avascular pancreatic islets. *Theoretical Biology and Medical Modelling* **6**, 5 (2009).

33 DeBerardinis, R. J., Lum, J. J., Hatzivassiliou, G. & Thompson, C. B. The biology of cancer: metabolic reprogramming fuels cell growth and proliferation. *Cell metabolism* **7**, 11–20 (2008).

34 Skog, S., Tribukait, B. & Sundius, G. Energy metabolism and ATP turnover time during the cell cycle of Ehrlich ascites tumour cells. *Experimental cell research* **141**, 23–29 (1982).

35 del Toro, R. *et al.* Identification and functional analysis of endothelial tip cell-enriched genes. *Blood*, blood–2010–2002–270819 (2010).

36 Jakobsson, L. *et al.* Endothelial cells dynamically compete for the tip cell position during angiogenic sprouting. *Nature cell biology* **12**, 943 (2010).

37 Kim, M.-C., Silberberg, Y. R., Abeyaratne, R., Kamm, R. D. & Asada, H. H. Computational modeling of three-dimensional ECM-rigidity sensing to guide directed cell migration. *Proceedings of the National Academy of Sciences* **115**, E390–E399 (2018).

38 Wong, B. W., Marsch, E., Treps, L., Baes, M. & Carmeliet, P. Endothelial cell metabolism in health and disease: impact of hypoxia. *The EMBO journal* **36**, 2187–2203 (2017).

39 Wood, L. B., Ge, R., Kamm, R. D. & Asada, H. H. Nascent vessel elongation rate is inversely related to diameter in in vitro angiogenesis. *Integrative Biology* **4**, 1081–1089 (2012).

40 Eichmann, A. & Simons, M. VEGF signaling inside vascular endothelial cells and beyond. *Current opinion in cell biology* **24**, 188–193 (2012).

41 Polacheck, W. J., Charest, J. L. & Kamm, R. D. Interstitial flow influences direction of tumor cell migration through competing mechanisms. *Proceedings of the National Academy of Sciences* **108**, 11115–11120 (2011).

42 Haessler, U., Teo, J. C., Foretay, D., Renaud, P. & Swartz, M. A. Migration dynamics of breast cancer cells in a tunable 3D interstitial flow chamber. *Integrative Biology* **4**, 401–409 (2011).
43 Nikmaneshi, M. R., Firoozabadi, B. & Mozafari, A. Chemo-Mechanistic multi-scale model of a three-dimensional tumor microenvironment to quantify chemotherapy response of cancer. *Biotechnology and Bioengineering* (2021).
44 Cameron, M. A. & Davis, A. L. A Mathematical Model of Angiogenesis in Glioblastoma Multiforme. (2009).
45 Nakatsu, M. N. *et al.* VEGF 121 and VEGF 165 regulate blood vessel diameter through vascular endothelial growth factor receptor 2 in an in vitro angiogenesis model. *Laboratory investigation* **83**, 1873–1885 (2003).
46 Netti, P. A., Roberge, S., Boucher, Y., Baxter, L. T. & Jain, R. K. Effect of transvascular fluid exchange on pressure–flow relationship in tumors: a proposed mechanism for tumor blood flow heterogeneity. *Microvascular research* **52**, 27–46 (1996).
47 Cai, Y., Zhang, J. & Li, Z. Multi-scale mathematical modelling of tumour growth and microenvironments in anti-angiogenic therapy. *Biomedical engineering online* **15**, 155 (2016).
48 Baish, J. W., Netti, P. A. & Jain, R. K. Transmural coupling of fluid flow in microcirculatory network and interstitium in tumors. *Microvascular research* **53**, 128–141 (1997).
49 Welter, M. & Rieger, H. Interstitial fluid flow and drug delivery in vascularized tumors: a computational model. *PloS one* **8**, e70395 (2013).
50 Bates, D., Hillman, N., Pocock, T. & Neal, C. Regulation of microvascular permeability by vascular endothelial growth factors. *Journal of anatomy* **200**, 523–534 (2002).
51 Cai, Y., Wu, J., Li, Z. & Long, Q. Mathematical modelling of a brain tumour initiation and early development: a coupled model of glioblastoma growth, pre-existing vessel co-option, angiogenesis and blood perfusion. *PloS one* **11**, e0150296 (2016).
52 DiResta, G. R. *et al.* Cell proliferation of cultured human cancer cells are affected by the elevated tumor pressures that exist in vivo. *Annals of biomedical engineering* **33**, 1270–1280 (2005).
53 Shirinifard, A. *et al.* 3D multi-cell simulation of tumor growth and angiogenesis. *PloS one* **4**, e7190 (2009).
54 Wu, B., Zhang, B., Li, B., Wu, H. & Jiang, M. Cold and hot tumors: from molecular mechanisms to targeted therapy. *Signal transduction and targeted therapy* **9**, 274 (2024).
55 Xiao, Z. *et al.* (2023).
56 Kong, X. *et al.* Transforming the "cold" tumors to "hot" tumors: strategies for immune activation. *Biochemical Pharmacology*, 117194 (2025).
57 Ganjalıkhani-Hakemi, M., Yanikkaya Demirel, G., He, X. & Zeng, C. Vol. 15 1425136 (Frontiers Media SA, 2024).
58 Schoenberg, M. B. *et al.* Perivascular tumor-infiltrating leukocyte scoring for prognosis of resected hepatocellular carcinoma patients. *Cancers* **10**, 389 (2018).
59 Mu, L. *et al.* (2017).
60 Anderson, A. R. A hybrid mathematical model of solid tumour invasion: the importance of cell adhesion. *Mathematical medicine and biology: a journal of the IMA* **22**, 163–186 (2005).
61 Jain, R. K. Transport of molecules in the tumor interstitium: a review. *Cancer research* **47**, 3039–3051 (1987).
62 Laursen, T. & Kirk, J. Diffusion coefficients of carbon dioxide and glucose for a connective tissue membrane from individuals of various ages. *Journal of gerontology* **10**, 303–305 (1955).
63 Bao, Z. *et al.* High glucose promotes human glioblastoma cell growth by increasing the expression and function of chemoattractant and growth factor receptors. *Translational oncology* **12**, 1155–1163 (2019).
64 Higgins, C. Parameters that reflect the carbon dioxide content of blood. *Parameters* (2008).

65 Anderson, A. R. & Chaplain, M. Continuous and discrete mathematical models of tumor-induced angiogenesis. *Bulletin of mathematical biology* **60**, 857–899 (1998).
66 Miura, T. & Tanaka, R. In vitro Vasculogenesis Models Revisited-Measurementof VEGF Diffusion in Matrigel. *Mathematical Modelling of Natural Phenomena* **4**, 118–130 (2009).
67 Höfer, T., Krichevsky, O. & Altan-Bonnet, G. Competition for IL-2 between regulatory and effector T cells to chisel immune responses. *Frontiers in immunology* **3**, 268 (2012).
68 Ridley, A. J. *et al.* Chemokines form complex signals during inflammation and disease that can be decoded by extracellular matrix proteoglycans. *Science Signaling* **16**, eadf2537 (2023).
69 Ross, A. & Pompano, R. Diffusion of cytokines in live lymph node tissue using microfluidic integrated optical imaging. *Analytica chimica acta* **1000**, 205–213 (2018).
70 Kihara, T., Ito, J. & Miyake, J. Measurement of biomolecular diffusion in extracellular matrix condensed by fibroblasts using fluorescence correlation spectroscopy. *PloS one* **8**, e82382 (2013).
71 Brunner, P., Kiwitz, L., Li, L. & Thurley, K. Diffusion-limited cytokine signaling in T cell populations. *Iscience* **27** (2024).
72 Marshall, A., Celentano, A., Cirillo, N., McCullough, M. & Porter, S. Tissue-specific regulation of CXCL9/10/11 chemokines in keratinocytes: Implications for oral inflammatory disease. *PLoS one* **12**, e0172821 (2017).
73 Liu, C. *et al.* Cytokines: from clinical significance to quantification. *Advanced Science* **8**, 2004433 (2021).
74 Derynck, R., Turley, S. J. & Akhurst, R. J. TGFβ biology in cancer progression and immunotherapy. *Nature reviews Clinical oncology* **18**, 9–34 (2021).
75 Lotze, M. T. *et al.* In vivo administration of purified human interleukin 2. II. Half life, immunologic effects, and expansion of peripheral lymphoid cells in vivo with recombinant IL 2. *The Journal of Immunology* **135**, 2865–2875 (1985).
76 Jiang, T., Zhou, C. & Ren, S. Role of IL-2 in cancer immunotherapy. *Oncoimmunology* **5**, e1163462 (2016).
77 Li, R. & Lei, J. Optimal therapy schedule of chimeric antigen receptor (CAR) T cell immunotherapy. *Mathematical Biosciences and Engineering* **22**, 1653–1679 (2025).
78 Baldwin, M. E. *et al.* The specificity of receptor binding by vascular endothelial growth factor-d is different in mouse and man. *Journal of Biological Chemistry* **276**, 19166–19171 (2001).
79 McIlhenny, C., George, W. & Doughty, J. A comparison of serum and plasma levels of vascular endothelial growth factor during the menstrual cycle in healthy female volunteers. *British journal of cancer* **86**, 1786–1789 (2002).
80 Billy, F. *et al.* A pharmacologically based multiscale mathematical model of angiogenesis and its use in investigating the efficacy of a new cancer treatment strategy. *Journal of theoretical biology* **260**, 545–562 (2009).
81 Davis, S. *et al.* Isolation of angiopoietin-1, a ligand for the TIE2 receptor, by secretion-trap expression cloning. *Cell* **87**, 1161–1169 (1996).
82 Longstaff, C. Plasminogen activation on the cell surface. *Front. Biosci* **7**, d244–d255 (2002).
83 Maisonpierre, P. C. *et al.* Angiopoietin-2, a natural antagonist for Tie2 that disrupts in vivo angiogenesis. *Science* **277**, 55–60 (1997).
84 Plank, M., Sleeman, B. & Jones, P. A mathematical model of tumour angiogenesis, regulated by vascular endothelial growth factor and the angiopoietins. *Journal of theoretical biology* **229**, 435–454 (2004).
85 Engin, H., Üstündağ, Y., Tekin, İ. Ö. & Gökmen, A. Plasma concentrations of Ang-1, Ang-2 and Tie-2 in gastric cancer. *European cytokine network* **23**, 21–24 (2012).

86 Cai, Y., Xu, S., Wu, J. & Long, Q. Coupled modelling of tumour angiogenesis, tumour growth and blood perfusion. *Journal of Theoretical Biology* **279**, 90–101 (2011).

87 Masuhara, K., Nakai, T., Yamaguchi, K., Yamasaki, S. & Sasaguri, Y. Significant increases in serum and plasma concentrations of matrix metalloproteinases 3 and 9 in patients with rapidly destructive osteoarthritis of the hip. *Arthritis & Rheumatism* **46**, 2625–2631 (2002).

88 Woodley-Cook, J. *et al.* Effects of the chemotherapeutic agent doxorubicin on the protein C anticoagulant pathway. *Molecular cancer therapeutics* **5**, 3303–3311 (2006).

89 Pries, A., Secomb, T. & Gaehtgens, P. Structural adaptation and stability of microvascular networks: theory and simulations. *American Journal of Physiology-Heart and Circulatory Physiology* **275**, H349–H360 (1998).

90 Stéphanou, A., McDougall, S. R., Anderson, A. R. & Chaplain, M. A. Mathematical modelling of the influence of blood rheological properties upon adaptative tumour-induced angiogenesis. *Mathematical and Computer Modelling* **44**, 96–123 (2006).

91 Stylianopoulos, T. & Jain, R. K. Combining two strategies to improve perfusion and drug delivery in solid tumors. *Proceedings of the National Academy of Sciences* **110**, 18632–18637 (2013).
